# Biochemical and hematological parameters (including thromboelastography) differ in patients with sepsis and SIRS after esophagectomy

**DOI:** 10.1186/cc9863

**Published:** 2011-03-11

**Authors:** M Durila, J Bronský, T Haruštiak, A Pazdro, M Pechová, K Cvachovec

**Affiliations:** 1 Department of Anesthesiology and Critical Care Medicine, Second Faculty of Medicine, Charles University, Prague, Czech Republic; 2 Department of Paediatrics, Charles University, Second Faculty of Medicine, Prague, Czech Republic; 3 Third Department of Surgery, First Faculty of Medicine, Prague, Czech Republic; 4 Department of Clinical Biochemistry and Pathobiochemistry, Second Faculty of Medicine, Charles University, Prague, Czech Republic

## Introduction

Early diagnosis of sepsis and its differentiation from non-infective SIRS is very important. The links between inflammation and coagulation play an important role in the SIRS/sepsis process. We investigated hematological and biochemical parameters (including thromboelastography (TEG)) in patients after surgical resection of esophagus. The aim of our project was to find out whether there are any changes in these parameters that could help in differentiation between SIRS and sepsis.

## Methods

In our study we enrolled 38 patients (aged 41 to 74) undergoing esophagectomy. Blood samples were obtained in the morning before the operation and then every 24 hours for the next 6 postoperative days (POD). Blood samples were analysed for the following parameters: procalcitonin (PCT), C-reactive protein (CRP), IL-6, aspartate transaminase (AST), lactate, white blood count (WBC), D-dimers, antithrombin (AT), international normalised ratio (INR), activated partial thromboplastin time (APTT) and parameters of TEG.

## Results

Nine patients developed sepsis within 6 postoperative days. Five of them had pneumonia and in four patients the cause of sepsis was dehiscention of gastroesophageal anasthosmosis. Significant differences between patients with SIRS and patients with sepsis were found in the following parameters: 0-day (before operation): no significant differences; POD 1: differences in AST (*P *< 0.002) only; POD 2: AST (*P *< 0.003), lactate (*P *< 0.006), D-dimers (*P *< 0.02), PCT (*P *= 0.03), IL-6 (*P *< 0.03), WBC (*P *< 0.03); POD 3: AST (*P *< 0.03), PCT (*P *< 0.02), IL-6 (*P *= 0.006), CRP (*P *< 0.04), WBC (*P *< 0.05); POD 4: AST (*P *= 0.006), PCT (*P *= 0.007), IL-6 (*P *< 0.02), CRP (*P *= 0.03), D-dimers (*P *< 0.05), INR (*P *= 0.03); POD 5: PCT (*P *< 0.003), IL-6 (*P *< 0.04), CRP (*P *< 0.04), AT (*P *= 0.03); and POD 6: PCT (*P *= 0.0001), CRP (*P *< 0.013), WBC (*P *= 0.03), TEG-LY30 (*P *< 0.04).

## Conclusions

Sequential measurement of biochemical and hemato-logical parameters, mainly AST, PCT, IL-6, WBC, CRP and D-dimers, can help in early diagnosis of sepsis in patients after extensive operation such as esophagectomy. On the contrary, TEG does not seem to be helpful in differentiation of SIRS/sepsis during the early postoperative period. However, it seems to be useful after the fifth postoperative day.

